# Sensor dimer disruption as a new mode of action to block the IRE1-mediated unfolded protein response

**DOI:** 10.1016/j.csbj.2022.03.029

**Published:** 2022-03-29

**Authors:** Kosala N. Amarasinghe, Diana Pelizzari-Raymundo, Antonio Carlesso, Eric Chevet, Leif A. Eriksson, Sayyed Jalil Mahdizadeh

**Affiliations:** a Department of Chemistry and Molecular Biology, University of Gothenburg, 405 30 Göteborg, Sweden; b INSERM U1242, Université de Rennes, Rennes, France; c Centre de Lutte contre le Cancer Eugène Marquis, Rennes, France; d Università della Svizzera italiana (USI), Faculty of Biomedical Sciences, Euler Institute, Lugano, Switzerland

**Keywords:** IRE1α, UPR, Peptide docking, Dimer disruptor, FDA approved drugs, MD simulations

## Abstract

The unfolded protein response (UPR) is activated to cope with an accumulation of improperly folded proteins in the Endoplasmic reticulum (ER). The Inositol requiring enzyme 1α (IRE1α) is the most evolutionary conserved transducer of the UPR. Activated IRE1 forms ‘back-to-back’-dimers that enables the unconventional splicing of X-box Binding Protein 1 (XBP1) mRNA. The spliced XBP1 (*XBP1s)* mRNA is translated into a transcription factor controlling the expression of UPR target genes. Herein, we report a detailed *in silico* screening specifically targeting for the first time the dimer interface at the IRE1 RNase region. Using the database of FDA approved drugs, we identified four compounds (neomycin, pemetrexed, quercitrin and rutin) that were able to bind to and distort IRE1 RNase cavity. The activity of the compounds on IRE1 phosphorylation was evaluated in HEK293T cells and on IRE1 RNase activity using an *in vitro* fluorescence assay. These analyzes revealed sub-micromolar IC_50_ values. The current study reveals a new and unique mode of action to target and block the IRE1-mediated UPR signaling, whereby we may avoid problems associated with selectivity occurring when targeting the IRE1 kinase pocket as well as the inherent reactivity of covalent inhibitors targeting the RNase pocket.

## Graphical abstract

**Figure:**
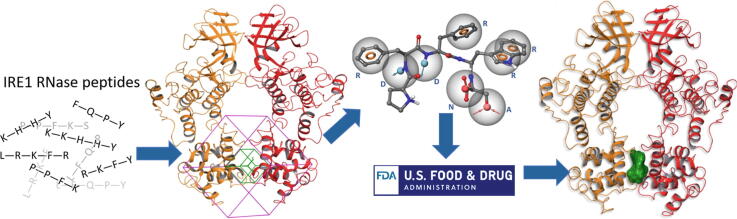

## 1 Introduction

The endoplasmic reticulum (ER) is a cellular organelle that comprises a network of elongated tubules and flattened discs and that can account for more than half of the total membrane surface area of the cell [1]. The unfolded protein response (UPR) is an adaptive cellular response to ER stress [2], and aims at restoring ER homeostasis [3]. UPR signaling is activated in response to the accumulation of improperly folded proteins in the ER lumen [4]. The UPR is transduced by three ER resident transmembrane sensors that trigger a series of downstream signaling events leading to enhanced expression of components of the ER protein folding, quality control and degradation machineries [2]. If ER stress cannot be resolved, the UPR triggers pro-apoptotic mechanisms [5].

**Fig. 1:**
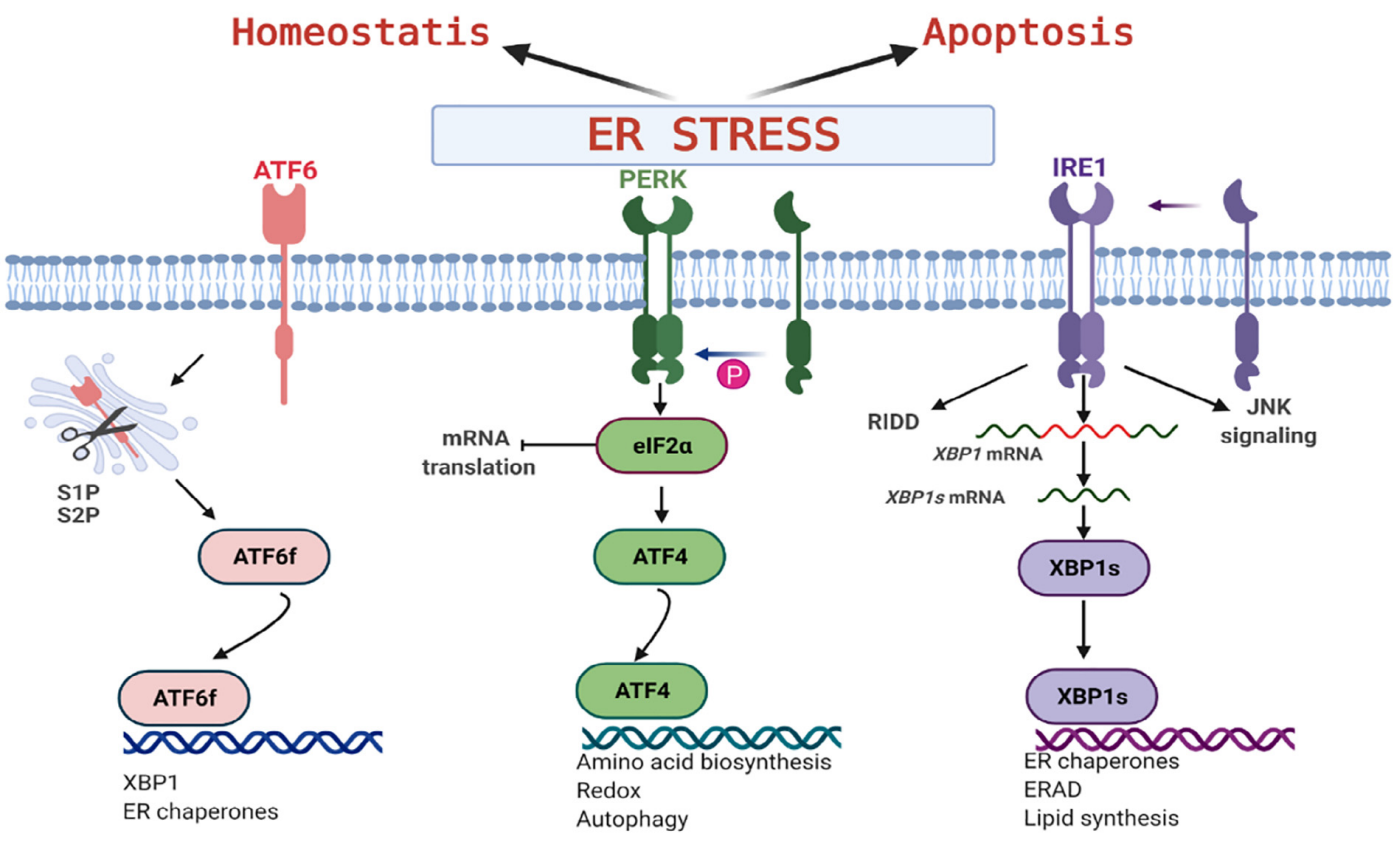
Simplified diagram of the core elements of the UPR signaling network. ER stress activates the stress sensors IRE1, PERK, and ATF6 representing the three branches of the UPR. Graph created with Biorender.com.

The three transmembrane ER-resident proteins responsible for transducing UPR signals from the ER lumen to the cytosol are the Inositol requiring enzyme 1α (IRE1α), the PKR–like ER kinase (PERK) and the Activating transcription factor 6 (ATF6) (Fig. 1) [5]. The UPR is involved in the development of many pathologies such as cancer, diabetes, inflammatory and degenerative diseases [5]. IRE1α (referred to as IRE1 hereafter), the most evolutionary conserved of the three sensors, contains an N-terminal luminal domain, a transmembrane domain, and cytoplasmic C-terminal kinase and endoribonuclease effector (RNase) domains [5]. Under basal conditions, IRE1 is maintained in an inactive monomeric state through the binding of its luminal domain to the ER chaperone BiP. During ER stress, BiP detaches from the IRE1 luminal domain, thus enabling the cytosolic domains to undergo *trans*-autophosphorylation and cooperative oligomerization, leading to activation of the RNase domain through the ‘back-to-back’ IRE dimers (Fig. 1) [6], [7]. IRE1 RNase is capable of cleaving X-box binding protein 1 (*XBP1*) mRNA, thereby removing a 26-nucleobase intron. The remaining exons are ligated by the tRNA ligase RtcB, generating the spliced *XBP1 (XBP1s*) mRNA which is translated into the active transcription factor XBP1s [6]. XBP1s induces the expression of genes whose products aim at restoring ER homeostasis [5]. In the second activation process known as regulated IRE1-dependent decay (RIDD) of RNA, IRE1 instead cleaves a number of RNA transcripts and micro-RNAs [8].

The profound role of IRE1 signaling on a wide variety of human diseases has resulted in a significant interest in identifying small molecule modulators either as activators or inhibitors [9], [10]. As such, several IRE1 inhibitors have been developed which either interact with the IRE1 RNase or kinase domains [11], [12]. Two types of ATP competitive ligands are known to bind reversibly to the IRE1 kinase active site: compounds that inhibit the kinase domain and allosterically activate the RNase domain, and inhibitors that inactivate both the kinase and RNase domains through their binding to the kinase site [11]. Inhibitors targeting the RNase site are to date all based on hydroxyl aryl aldehyde (HAA) moieties binding covalently to lysine 907 via a Schiff base mechanism [13]. No inhibitors have thus far been reported that explicitly target the dimer interface of the RNase domains, aiming to distort the dimer structure and thereby disrupt *XBP1* from binding properly to the RNase pocket.

**Fig. 2:**
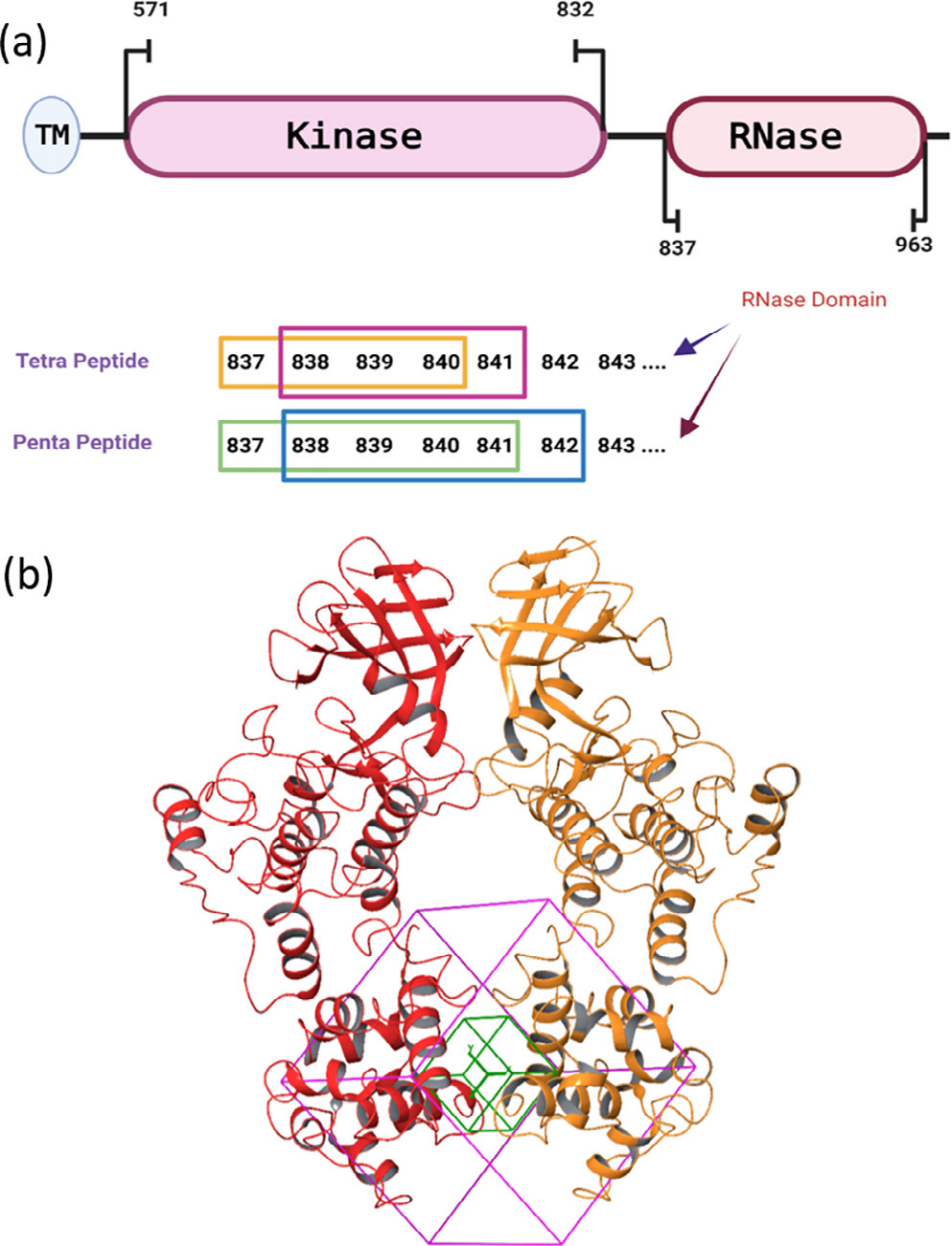
(a) The amino acid sequence of the *h*IRE1 RNase domain sequentially dissected to produce libraries of tetra- and penta-peptides. (b) Grid box used in the docking towards the dimer interface of the RNase domain in the *h*IRE1 back-to-back dimer.

In previous work, peptide fragments from the human IRE1 (*h*IRE1) kinase domain were identified that efficiently could inhibit IRE1 activity by binding to the kinase pocket [14], [15]. Pharmacophores based on the smaller peptides led to the identification of Food and Drug Administration (FDA) approved drugs inhibiting IRE1 activity and showing good activity in sensitizing glioblastoma cells to chemotherapy [15]. Herein, a similar route was taken to characterize promising peptide fragments derived from the IRE1 RNase domain (Fig. 2a) that could target the IRE1 back-to-back dimer interface region at the RNase site, and from this identify small molecules that might have high affinity towards the same areas. Using different *in silico* techniques such as peptide and molecular docking, molecular dynamics (MD) simulations, and pharmacophore analyses we were able to identify four compounds from the database of FDA approved drugs that bind to the dimer interface and distort the RNase site. In subsequent *in vitro* fluorescence assays designed to measure IRE1 RNase activity, these were confirmed as good modulators, with sub-micromolar IC_50_ values. The key findings were also verified through analysis of IRE1 phosphorylation upon treatment of HEK293T cells with the different inhibitors. This was achieved using immunoblotting following protein resolution on PhosTag gels. This is the first reported study identifying compounds blocking the UPR by directly distorting the IRE1 dimer, and opens for new therapeutic modes of action targeting, *e.g.,* cancer or degenerative diseases.

## 2 Methods

### 2.1 Protein preparation

The human IRE1 back-to-back crystal structure with PDB code 4YZC (Staurosporine bound to the kinase domain) [16] was downloaded from the protein data bank and prepared using the Schrodinger protein preparation wizard (*i.e.* assign bonds and bond orders, completing missing loops or side chains using Prime) [17], [18], [19]. After fixing structural defects, water molecules were removed from the system. The hydrogen bonding network was optimized by adjusting the protonation states of Asp, Glu and tautomeric states of His to match a pH of 7.0 ± 2 [20], [21]. Finally, the IRE1 dimer was subjected to geometry refinement using the OPLS3e force field in restrained structural minimization [22], [23], [24], [25].

### 2.2 Peptide library

The tetra- and penta-peptide libraries were built using the sequence of the *h*IRE1 RNase domain as shown in Fig. 2, totaling 286 unique sequential tetra- and penta-peptides covering all the IRE1 RNase domain residues. Finally, each peptide was subjected to the protein preparation process as described above [17], [20].

### 2.3 Peptide docking

Peptide docking was performed using the Glide peptide-docking tool in Schrödinger [26]. Using the grid docking generator, the binding site was defined as the centroid of the IRE1 RNase dimer region comprising residues 825–977 from each monomer. The generated cubic grid box (with the dimension of 30 Å) is shown in Fig. 2b. All other parameters were set to the default values according to the Glide docking process. The special peptide docking mode of Glide is designed to handle the much greater flexibility of peptides relative to the usual kinds of small molecule ligands though improved sampling, and enabled the analysis of more than 23,000 conformers in the current study.

### 2.4 Pharmacophore modeling

The peptide poses with the highest docking scores were selected to developed three-dimensional pharmacophore hypotheses using the Phase software in Schrödinger [27], [28]. The pharmacophore hypotheses were generated based on complementary features in the receptor-peptide complexes using the e-Pharmacophore method along with excluded volume defined as regions of space occupied by the receptor [29], [30]. The e-Pharmacophore technique employs the Glide extra precision (XP) scoring function to accurately identify protein-peptide interactions, resulting in improved database screening enrichments [29], [30].

### 2.5 FDA dataset virtual screening and docking

The pharmacophore hypotheses generated from the best peptide docking poses were used to screen the database of FDA-approved drugs (version Sept. 2020) identifying small molecules with similar binding capabilities as the peptides. For compounds to be defined as hits in the pharmacophore-based virtual screening, these were required to match at least 4 pharmacophore points in a model. The pharmacophore tolerance value was set to 2.0 Å, and 50 different conformers were generated and minimized for each compound during the search.

The screening outcomes were then docked into the IRE1 RNase dimer binding site using Glide in Schrödinger [31], [32], [33]. The binding site and grid used for the FDA-approved ligands was the same as in the peptide docking. All other parameters were set to defaults. All molecular docking calculations were conducted using Glide extra precision (XP) along with the OPLS3e force field [24], [31].

### 2.6 MD simulations

A series of MD simulations were carried out to investigate the overall stability of the best docked FDA candidates in the RNase binding site of the IRE1 back-to-back dimer. The MD simulations were performed using the Desmond MD engine, an explicit-solvent molecular dynamics program implemented in the Schrödinger package [34]. The TIP3P water model was used to simulate water molecules in an orthorhombic box positioned such that the walls were at a minimum 10 Å distance from any atoms of the system [35]. The biological salt concentration 0.15 M was considered and counter ions (*i.e.,* Na^+^/Cl^-^) were added to balance the system charge. The default Desmond protocol was performed for minimization and relaxation prior to the start of the simulations [34]. Periodic boundary conditions and the OPLS3e force field were applied in the MD simulations [24], in which Nose-Hoover temperature coupling and Martyna–Tobias–Klein barostat [36], [37] were employed to keep the temperature and pressure kept constant at 300 K and 1 atmospheric pressure, respectively, in an NPT ensemble. Following equilibration, the MD simulations were run for 100 ns with a trajectory sampling frequency of 100 ps in the production steps.

### 2.7 *In vitro* IRE1 RNase assay

The assay was performed as described previously [38]. Briefly, each drug was diluted in minimal volume of solvents, following their datasheets (Quercitrin – Ref. Y0001931 Merck® and Rutin - Ref. R5143 Merck® in DMSO, Premetexed - Ref. Y0001539 Merck® and Neomicin - Ref. N6386 Merck® in water 0.9% NaCl). Subsequently each compound was re-diluted in reaction buffer (20 mM HEPES-NaOH pH 7.5; 1 mM MgOAc; 50 mM KOAc). Maximum volume of solvent per reaction never exceeded 1%. Reaction volume was 25 μl. In each reaction, 0.6 μg of recombinant IRE1 (aa 465–977, His & GST Tag, SinoBiological®) was incubated at room temperature for 10 min with varying concentrations (0–10 μM) of each compound and reaction buffer. The assay relied on the quenched emission of fluorescence mini IRE1 RNA substrate probe (5′- CAUGUCCGCAGCGCAUG-3′; Eurogentec®), which when cleaved by IRE1 emits fluorescence at 590 nm (cy5) wavelength [39]. Equal volumes of a mixture of reaction buffer, 10 mM ATP, 1 mM DTT and 1 μg of fluorescent probe were added to each sample and fluorescence was read in 96 well plates flat bottom, black polystyrene, matrix active group High Bind (Corning®) every minute for 25 min, at 37 °C, using a Tecan 200 plate reader.

### 2.8 Phosphorylation assay

***Cell culture* –** HEK293T cells were tested for the absence of mycoplasma using MycoAlert® (Lonza, Basel, Switzerland) or MycoFluor (Invitrogen, Carlsbad, CA, USA). HEK cells were grown in DMEM Glutamax (Invitrogen, Carlsbad, CA, USA) supplemented with 10% FBS in a 5% CO_2_ humidified atmosphere at 37 °C.

***Transfection* -** Cells were seeded on 6-well plates at 2 × 10^6^ cells/mL concentration and incubated overnight. On the following day the cells were transfected with WT IRE1 plasmid with Lipofectamine® LTX Reagent (Thermofisher®) as described in the manufacturer's protocol.

***Treatments*** - Transfected cells were permeabilized with Saponin (0.001%) for 30 min at 37 °C/ 5% CO_2_ to allow the internalization of the compounds. Each compound (neomycin, pemetrexed, rutin and quercitrin) was treated for 1 h at 25 µM. Tunicamycin was used at 1 μg/mL for 1 h. After the incubation time, cells were lysed with RIPA lysis buffer at 4 °C for 25 min. Total protein was quantified using Pierce™ Rapid Gold BCA Protein Assay Kit (Thermofisher®) and 10 μg immediately loaded on SuperSep Phos-tag Zn^2+^ gels.

***Western Blot* -** SuperSep Phos-tag (50 µmol/L)™ with Zn^2+^(Interchim®; Ref.1H6280.193–16571) were used to analyze IRE1 phosphorylation. The running buffer for Zn^2+^ consisted of 25 mM Tris-Cl pH 7.4, 192 mM glycine, SDS 1%. The power supply settings were adjusted for optimal separation of phosphorylated and non-phosphorylated protein species. For this reason, all gels run at 10–15 mA/gel constant current and electrophoresis required 6–8 h for completion at 4 °C. Gels were pretreated with washing in transfer buffer with EDTA (25 mM Tris-Cl pH 7.4, 192 mM glycine, 10 mM EDTA) for 3 times 20 min each in order to remove bivalent cations that would immobilize phosphorylated proteins in the gel not allowing their transfer to the nitrocellulose membrane. Finally, the gel was equilibrated for 10 min in transfer buffer without EDTA. The transfer of the proteins from the gel to the membraned was performed with transfer buffer containing 10% v/v ethanol at 100 V for 1 h. For efficient transfer of Phos-Tag™ gel separated proteins wet-tank transfer was used. The membranes were stained with 3% w/v Ponceau S in 5% v/v aqueous solution of acetic acid to validate transfer efficiency. Subsequently membranes were thoroughly destained with milliQ water and TBST (10 mM Tris–HCl (pH 7.5), 100 mM NaCl, and 0.10% v/v Tween-20). Non-specific antibody binding was blocked by incubating membranes in 5% w/v BSA in TBST for 1 h. All IRE1 signaling analyses were carried out as described previously [40]. IRE1 total and phosphorylated forms were stained using anti‐IRE1 antibody (Anti-human; CellSignalling®, IRE1α (14C10) Rabbit mAb#3294) Tubulin was used as a loading control (Sigma®, T5168). The membranes were incubated with ECL reagent (ECL RevelBlOt® Intense, Ozyme) according to manufacturer's instructions. Immunoreactive bands were documented with a Genesys™ in a GBox System. Prestained MW markers migrate anomalously in Phos-Tag™ gels. Due to that Western Blots of those gels cannot be assigned using the standard protein ladders. As such, protein identity is based on known immunoreactivity.

### 2.9 Statistical analyses

Fluorescence data are presented as mean ± SEM. Statistical significance (*P* < 0.05 or less) was determined using unpaired *t*‐tests or ANOVA as appropriate and, along with curve extrapolations, performed using GraphPad Prism software (GraphPad Software, San Diego, CA, USA).

### 2.10 Data availability

Datasets with input files, protein-peptide and ligand docking datasets and simulation trajectories are freely available at zenodo.org as https://doi.org/10.5281/zenodo.4050117.

## 3 Results

### 3.1 Peptide and pharmacophore modeling

The IRE1 RNase peptide library consisting of a total of 286 tetra- and penta-peptides was docked into the RNase domain of the *h*IRE1 back-to-back dimer using the Glide peptide docking tool available in Schrödinger [26]. To avoid any bias, the docking grid was designed to include all the RNase residues in the dimer structure, centered at the monomer–monomer RNase domain interface mid-point; *cf* Fig. 2b. The output of the peptide docking consisted of 23,394 conformers of the tetra- and penta-peptides bound to the RNase domain of the *h*IRE1 dimer. Peptides with docking score better than −11.3 kcal mol^−1^ were chosen for further analysis (Fig. S1). Table S1 shows the best four peptide sequences along with their docking scores and free energy of binding values, *ΔG_b_*, calculated using the MM-GBSA approach [41]. The best peptides were PFFWS, LRKFR and LFQPY (*h*IRE1 residues 830–834, 886–890 and 956–960, respectively), all with docking score −11.4 kcal/mol, and KKHHY (*h*IRE1 residues 907–911) with docking score −12.2 kcal/mol. The large negative values for the docking scores and free energies of binding indicate high affinity of these peptides towards the RNase domain of the human IRE1 back-to-back dimer.

**Fig. 3:**
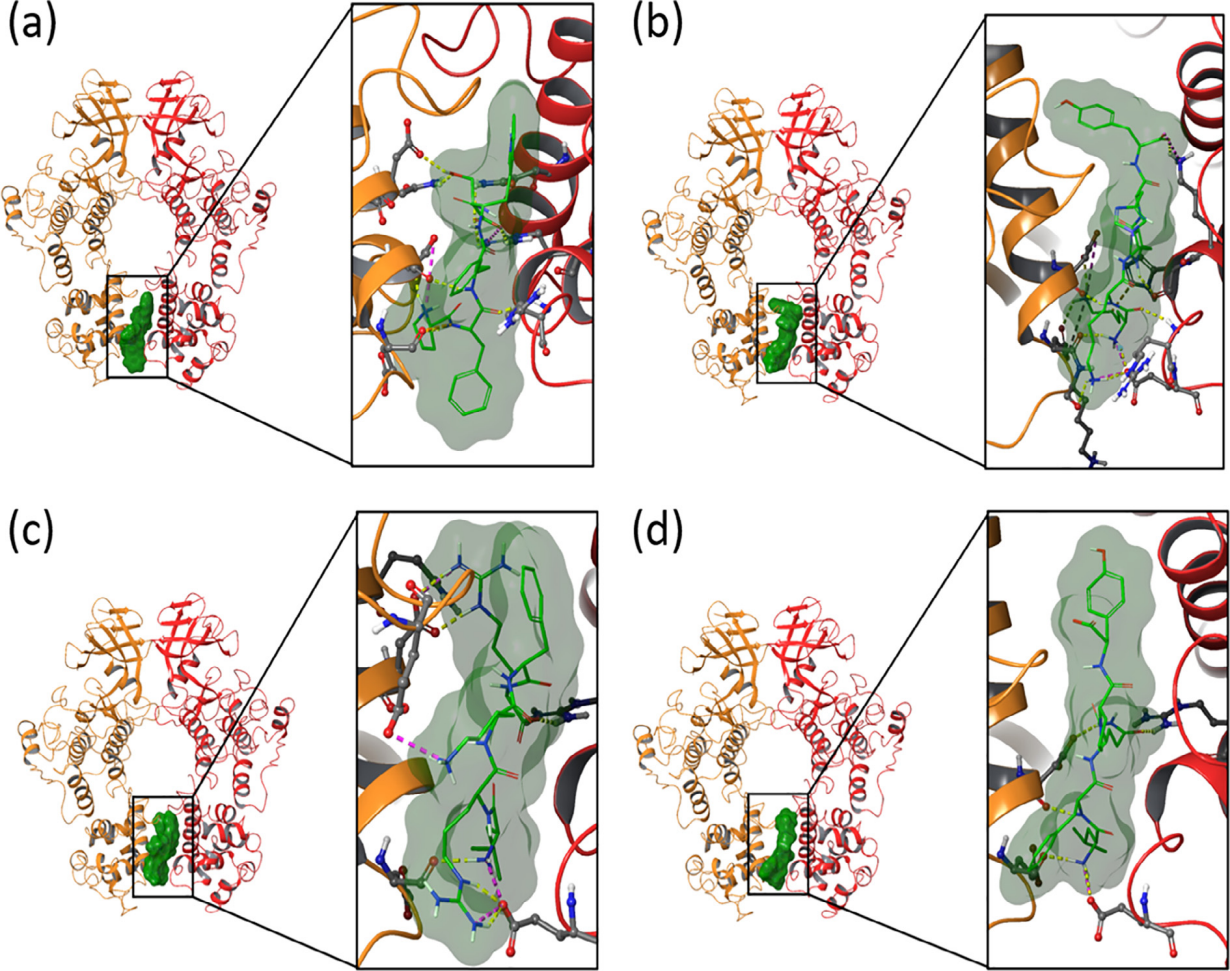
Binding site overview for (a) PFFWS, (b) KKHHY, (c) LRKFR, and (d) LFQPY peptides bound to the RNase domain interface of the human IRE1 back-to-back dimer. Yellow and pink dashed lines represent hydrogen bonds and salt bridge interactions, respectively. (For interpretation of the references to colour in this figure legend, the reader is referred to the web version of this article.)

The best docking pose for each of the four peptides bound to the RNase domain along with atomic contacts is shown in Fig. 3. The 2D interaction diagrams of the same IRE1-peptide complexes, within a 4 Å cutoff radius from the ligand, are presented in Fig. S2. The best binding peptide, KKHHY, forms ten hydrogen bonds (A:Lys908, A:His909, A:Arg912, A:Glu913, B:Asp847, B:Glu850, and B:Lys851) and three salt bridges (A:Lys908, A:Glu913, and B:Glu850). The PFFWS peptide forms eight hydrogen bonds (through residues A:Gln843, A:Arg905, A:Arg912, B:Gln843, B:Asp844, B:Asp847, and B:Glu850) and two salt bridges (through residues A:Arg905 and B:Asp847). LRKFR forms six hydrogen bonds (A:Gln840, A:Glu913, B:Asp844, and B:Glu850) and four salt bridges (A:Arg905, A:Glu913, B:Asp844, and B:Glu954). LFQPY, finally, forms six hydrogen bonds (A:Arg905, A:Arg912, A:Glu913, B:Asp847, and B:Glu850) and one salt bridge (A:Glu913). Based on this IRE1-peptide interaction analysis, we note that Arg905, Arg912, Asp847, Glu850, Glu913, and Gln843 are the most conserved residues with highest contributions to the peptide binding.

Pharmacophore hypotheses were developed for the highest scoring docking poses, representing peptides PFFWS, KKHHY, LRKFR, and LFQPY. The pharmacophore hypotheses were generated based on the complementary features in the receptor-peptide complexes using the e-Pharmacophore method along with the associated excluded volume [29], [30]. Fig. S3 shows the pharmacophore hypotheses superposed on the best pose of each docked peptide.

The different points in the pharmacophore models represent hydrogen bond acceptor (A), hydrogen bond donor (D), hydrophobic site (H), negative ionic site (N), positive ionic site (P), and aromatic ring (R). The PFFWS peptide pharmacophore model includes 8 points in the form of ADDNRRRR in which A is located on side chain of Ser834 (number of the peptide residues as in the IRE1 sequence), D are on backbone nitrogen atoms of Phe832 and Phe832, N is on the C-terminus, and the R points are on the side chains of Phe832, Phe832 and Trp833. The best-scoring KKHHY peptide model pharmacophore consists of seven points in the form of ADDNRRR in which A is located on oxygen atom of carbonyl backbone of Lys907, D are on backbone nitrogen of Lys907 and side chain of Tyr911, N is the C-terminus, and the R points are on the side chains of His909, His910, and Tyr911. The LRKFR peptide model pharmacophore includes four points in the form of DDNR in which the D points are located on the side chain of Arg887 and Arg890, N is on the C-terminus, and R is located on the side chain of Phe889. The LFQPY peptide pharmacophore hypothesis consists of six points in the form of AADDRR in which the A points are located on the oxygen atom of the backbone carbonyl of Leu956 and the side chain of Gln958, the D points are on the backbone nitrogen of Phe957 and side chain of Gln958, and R points are on the side chains of Phe957 and Tyr960.

### 3.2 Virtual screening of FDA approved drugs

**Fig. 4:**
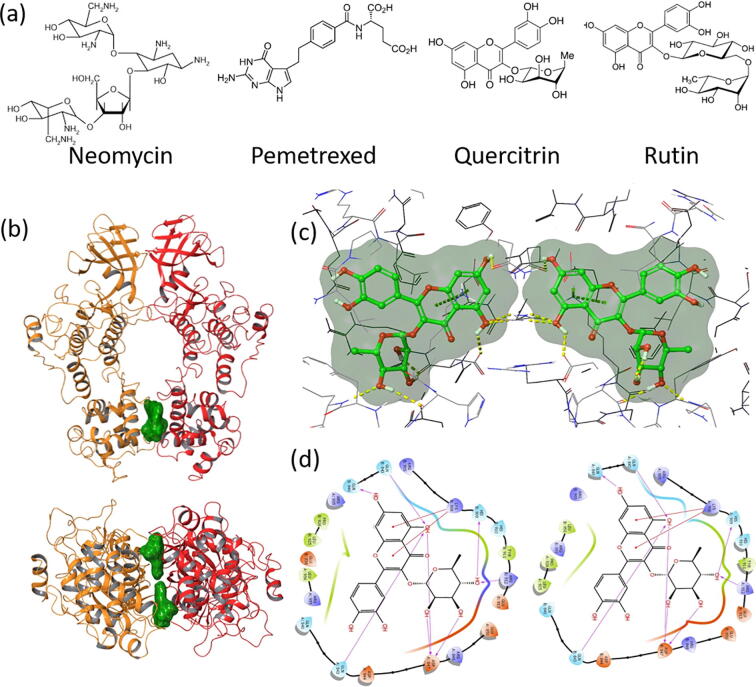
(a) The four best docked compounds neomycin, pemetrexed, quercitrin and rutin identified in the current study. (b) 3D views of two molecules of quercitrin bound to the RNase domain of IRE1 dimer; top: front view; bottom: RNase domain viewed from below. One IRE1 monomer is depicted in red, and the other in orange. (c) The corresponding atomic contacts in 3D. (d) 2D interaction diagrams. The corresponding data in (b)-(d) for neomycin, pemetrexed and rutin is displayed in Fig. S4. (For interpretation of the references to colour in this figure legend, the reader is referred to the web version of this article.)

The four pharmacophore hypotheses generated from the peptidomimetic study were subsequently used to screen the database of FDA-approved drugs (version Sept 2020; ∼2700 compounds) to identify small molecules with similar binding capabilities as the IRE1 RNase derived peptides. All the hits identified from the pharmacophore-based screening were docked into the RNase binding pocket of the IRE1 back-to-back dimer as depicted in Fig. 2b, using Glide and XP scoring function. The four compounds neomycin, pemetrexed, quercitrin and rutin (Fig. 4a) were identified by filtering off hits with docking scores less than −8.0 kcal/mol. Interestingly, all four molecules resulted from the screening based on the pharmacophore obtained with the PFFWS peptide (residues 830–834) which in IRE1 is located at the linker region between the kinase and RNase domains. Based on the position in the binding site and symmetric structure of the IRE1 dimer, two copies of each molecule could be docked into the RNase domain. The binding site position along with atomic contacts between IRE1 and quercitrin are shown as 3D and 2D interaction diagrams in Fig. 4, and in Fig. S4 we present the same data for neomycin, pemetrexed and rutin. Table 1 shows the docking scores and free energies of binding calculated from molecular docking and MMGBSA calculations, respectively, for all four molecules.

**Table 1:** Docking score values and free energies of binding, ΔG_b_, for each hit identified from the virtual screening.

Compound^a^	From peptide	DockingScore (kcal mol^−1^)	ΔG_b_ (kcal mol^−1^)
Neomycin-1	PFFWS	−10.65	−34.3
Neomycin-2	PFFWS	−10.60	−32.5
Pemetrexed-1	PFFWS	−8.9	−37.7
Pemetrexed-2	PFFWS	−8.2	−36.5
Quercitrin-1	PFFWS	−12.4	−73.7
Quercitrin-2	PFFWS	−12.3	−58.7
Rutin-1	PFFWS	−11.9	−69.4
Rutin-2	PFFWS	−10.7	−64.6

^a)^1 and 2 refers to the two molecules bound to the RNase dimer region of the IRE1dimer.

As Fig. 4 and S4 shows, all four compounds dock well into the identified binding site. Due to the symmetry of the system, the interacting residues are identical for each of the two molecules in the respective complexes. Each quercitrin molecule interacts primarily with Arg912, Gln843, Gln840, Lys908, His909, and Asp847; pemetrexed with Arg912, Gln843, Gln840, and Arg905; rutin with Asp847, Gln843, Gln840, Asp844, Lys908, His909, Leu956, Glu836, Tyr911, Arg912, Leu925 and Glu850; and neomycin with Leu925, Gln840, Glu850, Ser924, Asp927, Leu956, Gln843, Glu836, Lys908, Asp844 and Asp847. Interaction with the two glutamines Gln840 and Gln843 are common between all four compounds, and interactions to Arg912 or Asp847 are common between three of the docked systems. Neomycin and rutin form interactions with the most residues; however, the best docking scores and highest free energy of binding is noted for quercitrin (Table 1).

The IRE1-ligand complexes obtained from molecular docking were the subjected to 100 ns MD simulations to confirm the overall stability of the hit compounds within the novel binding site. Fig. S5 depicts the RMSD and RMSF plots of the ligands and IRE1 dimers during the MD simulations. The RMSD curves show that the pemetrexed, quercitrin and rutin initially change their positions slightly inside the binding pocket during the MD simulation, but very rapidly reach stable conformations through favorable atomic interactions with surrounding residues. The one system that stands out as different is the IRE1-neomycin complex, which has significantly higher RMSD values for both the protein and the ligands. Similarly, the RMSF values are very low for all ligands except neomycin, with values close to 1 Å for most atoms in the other three ligands except, *e.g.,* the terminal carboxyl group of pemetrexed, or a few of the OH groups in rutin. The compound displaying the least movements inside the pocket is quercitrin, with RMSF values ∼ 1 Å for all atoms in both molecules.

Fig. S6 shows side-views of the first (t = 0) and last (t = 100 ns) snapshots of the complexes from the MD simulations. As the figures indicate, pemetrexed, quercitrin and rutin all remain in their initial binding cavities (*i.e.,* docked structures) with only slight movements in certain parts of the molecules. For neomycin, however, a large out-of-plane twisting movement away from the original ‘planar’ arrangement is noted for the RNase region of the dimer complex, in agreement with the large RMSD and RMSF data noted for this system. To further analyze the structural differences imposed by the ligands, we monitored the distances between the Cα atoms of His910 and Lys907 from one IRE1 monomer to the other during the MD simulations. His910 and Lys907 are residues essential for the catalytic cleavage reaction of the XBP1 mRNA [13]. The A:His910 – B:His910 and A:Lys907 – B:Lys907 distance evolutions during the MD simulations are shown in Figs. S7a and S7b, respectively, for the four complexes and the apo *h*IRE1 dimer (PDB-ID 4YZC). For the unperturbed system (crystal structure), the A:His910 – B:His910 and A:Lys907 – B:Lys907 distances are 17.1 and 20.9 Å, respectively (Fig. S7c). The average A:His910 – B:His910 and A:Lys907 – B:Lys907 distances over the last 50 ns of the MD trajectories increase to 19.4 and 22.5 Å, respectively. The complex with rutin displays similar values. Both the pemetrexed and quercitrin complexes, however, yield a steady and significantly wider gap between A:His910 – B:His910 and A:Lys907 – B:Lys907 with average distances of 23.3/24.4 Å (pemetrexed), and 23.3/26.4 Å (quercitrin) for the distance pairs, respectively. This information, together with the strong interaction and (from RMSF data) stable binding would indicate that the latter two molecules may be able to distort the RNase cavity and abrogate XBP1 binding. For neomycin, the twisting of the dimer away from the ‘planar’ structure is manifested through a dramatic decrease in A:His910 – B:His910 and A:Lys907 – B:Lys907 distances, to the average values of 10.2 and 19.1 Å, respectively, thereby exposing each half of the RNase cavity to the bulk solvent.

Figs. S8-S11 illustrate the abundance of protein–ligand atomic contacts during the MD simulations along with corresponding histogram and timeline interaction diagrams. There are many common residues interacting with the quercitrin, pemetrexed and rutin molecules, with high interaction abundancy (*i.e.*, Lys908, His909, Arg912, Asp844, Arg905, Leu956, Asp847, Gln840 and Gln843); Figs. S9-S11. As a consequence of the out-of-plane twisting in the neomycin complex, this displays significantly fewer interactions with IRE1 (Fig. S8), and mainly involves negatively charged residues forming salt bridges to the protonated amines. The high positive charge of neomycin was predicted during ligand preparation step to have the lowest state penalty (*i.e.,* the most abundant form at neutral pH).

### 3.3 *In vitro* IRE1 RNase assay

**Fig. 5:**
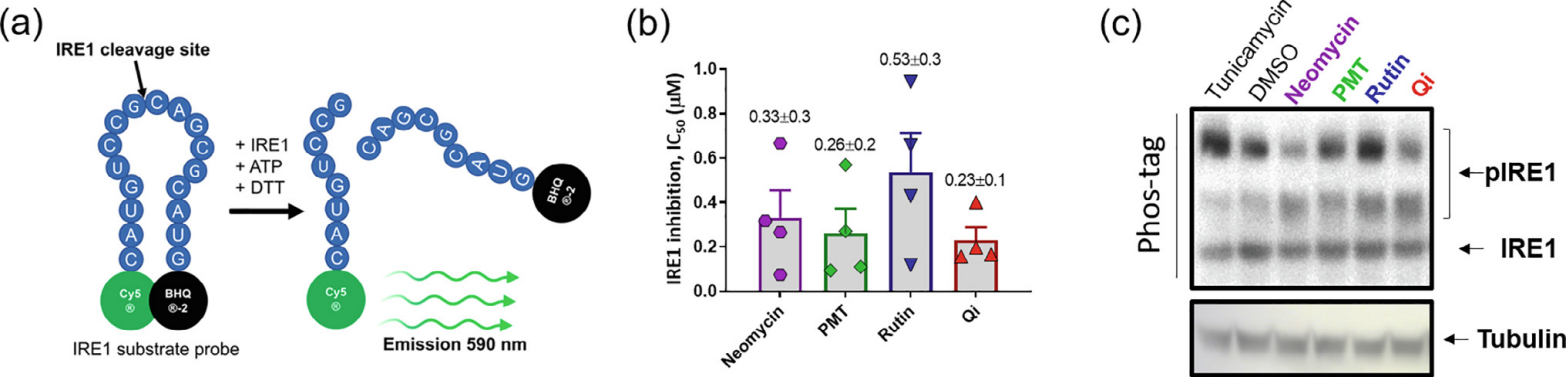
(a) Schematic representation of the IRE1 RNase activity *in vitro* assay. (b) IC_50_ values calculated from the fitting curves of IRE1 RNase activity performed in the presence of increasing concentrations of neomycin**,** pemetrexed (PMT), rutin and quercitrin (Qi) (Fig. S12). Fluorescence signals were detected as a read-out of RNA probe cleavage after 25-minute incubation. Symbols and error bars represent mean values ± SEM (c) Effect of the compounds on IRE1 phosphorylation status. Immunoblotting of IRE1 following protein resolution on PhosTag gels. Treatment with tunicamycin (1 µg/ml), DMSO, neomycin, pemetrexed (PMT), rutin and quercitrin (Qi) were performed on HEK cells transfected with WT IRE1 and permeabilized with Saponin (0.001%) for 1 h treatment (25 µM of each compound).

To test the validity of the obtained data an *in vitro* fluorescence assay measuring the cleavage of mRNA XBP1 mini-probe containing the consensus IRE1 cleavage site in a P-loop by *h*IRE1 cytosolic domain was used (Fig. 5a). All four compounds showed a clear inhibitory effect *in vitro*, with IC_50_ values well below 1 μM in all four cases (Fig. 5b). Best effect was observed for the flavonoid quercitrin with an IC_50_ value of 0.23 ± 0.1 μM. Quercitrin has previously been identified as an inhibitor of aldose reductase, and to have potential antiviral/antimicrobial activity towards Dengue virus, Leishmania, and HIV-1. The folate antimetabolite pemetrexed used in combination-treatment with cisplatin against pleural mesothelioma and non-small cell lung cancer was also a strong modulator of IRE1 RNase activity, with IC_50_ value of 0.26 ± 0.2. This was followed by the antibiotic neomycin with an IC_50_ of 0.33 ± 0.3 μM. The bioflavonoid rutin, which is a strong antioxidant, displayed the lowest inhibitory effect of the four with an IC_50_ just above 0.5 μM (0.53 ± 0.3 μM).

The better binding of quercitrin is in agreement with the findings from the *in silico* studies, whereas pemetrexed apparently is a significantly better inhibitor based on the *in vitro* data, than noted from the docking scores and free energies of binding (Table 1). Both compounds, however, resulted in a widening of the RNase pocket, and showed very stable binding towards the allosteric binding site during the MD simulations. The weakest inhibitory effect, noted for rutin out of the four compounds, can be rationalized by the very small distortions of the dimer structure for this particular compound (Fig. S7). Interestingly, all four compounds display as good as, or better, inhibitory effect in fluorescence assays than the covalent binder MKC8866 (IC_50_ = 0.78 ± 0.8 μM). MKC8866 is a hydroxyl aryl aldehyde that binds through Schiff base formation to Lys907 in the RNase binding pocket, thereby blocking the access for the *XBP1* mRNA substrate [42], [43], [44], [45], [46], [47], [48].

### 3.4 Phosphorylation assay

To further explore if the compounds also had an effect on XBP1 mRNA splicing in cells, transfected HEK293T cells were treated with 25 μM of either of the compounds for 1 h (for the ER stressor tumincamycin, 1 μg/mL was used). Following cell lysis, proteins were resolved using Phostag gels and then analyzed using immunoblot with antibodies against IRE1 (Fig. 5c). The immunoblots showed the effects of different treatments on IRE1 phosphorylation levels (pIRE1 bands). The ER stressor tunicamycin increased IRE1 hyperphosphorylation compared to the control (DMSO) whereas compounds such as neomycin and quercitrin decreased IRE1 phosphorylation levels (compared to DMSO). Pemetrexed (PMT) and, in particular, Rutin did not show any effect on IRE1 phosphorylation compared to the control, thus demonstrating no effect on IRE1 kinase activity (Fig. 5c). Given that the mechanism of activation of IRE1 dimers occurs through *trans*-autophosporylation, the low phosphorylation levels of quercitrin and neomycin can be attributed to the distortion of the dimers, thus rendering these less apt to engage in the autophosphorylation and subsequent *XBP1* mRNA splicing.

## 4 Conclusions

In the current study, we have identified the four FDA approved compounds neomycin, pemetrexed, quercitrin and rutin, as potential new modulators of human IRE1 using a combination of peptide docking, pharmacophore modeling, molecular docking, and classical MD simulation. The peptide library employed in this work was generated based on overlapping tetra- and penta-peptides derived from RNase domain of IRE1 (residues 837–977). From the peptide docking we identified a novel allosteric site within the RNase domain of the human IRE1 back-to-back dimer. This novel binding site did not correspond to the already known IRE1 druggable pockets such as the kinase pocket, the quercetin activator pocket found in yeast IRE1 dimers (*y*IRE1) [49], or the covalent binding sites of hydroxyl aryl aldehydes in the RNase pocket (Fig. S6f). Instead, the novel pocket is placed in the dimer interface region of the RNase domains in the dimer. Binding of ligands in this site resulted in a widening of the RNase pocket for two of the systems (quercitrin and pemetrexed), which also have the strongest binding and best IC_50_ values of the four. Rutin, albeit not yielding any significant distortion to the RNase pocket compared to the apo protein, nonetheless showed a non-negligible IC_50_, whereas neomycin resulted in an out-of-plane twisting of the dimer RNase regions. Of note, the *y*IRE1 dimer with two quercetin molecules bound [49], place these closer to the kinase domain than the currently identified binding site, and leads to activation – presumably by stabilizing the formed dimer and the RNase pocket through additional hydrogen bonded networks. Further work aiming to address this question is currently underway. Interestingly, quercitrin identified as an inhibitor to *h*IRE1 is a glycoside formed by the *y*IRE1 activator quercetin and the mono-saccharide rhamnose.

It is hypothesized that the conformational changes to the RNase pocket caused by the currently identified dimer disruptors, will either prevent *XBP1* mRNA binding to the RNase domain or disable its cleavage/splicing reaction and may thus consequently be able to block the genetic response to ER stress and trigger apoptosis. An advantage of targeting the dimer interactions region is that we avoid the issues of selectivity that arise when aiming to target the kinase pocket, as well as the problems associated with high/non-specific reactivity often encountered for covalent inhibitors. The findings herein, showing the existence and activity of IRE1 dimer disruptors, opens for an entirely new mode of action to block the UPR, and can serve as an additional and viable route to trigger apoptosis or sensitize cells in adjuvant therapies also including *e.g.* a cytotoxic compound. We also emphasize that the molecules thus identified from the database of FDA approved compounds, as such may display low selectivity towards IRE1 *in vivo* due to interactions with other possible targets. The low activity in the phosphorylation assays for pemetrexed and rutin in the current study are two such examples. However, as shown in the current work, they may constitute an important start for further medicinal chemistry studies to develop dimer disruptors.

## 5 Associated content

Table S1 and Figs. S1-S12 are available as Supplementary material to the current publication. Datasets with input files, protein-peptide and ligand docking datasets and simulation trajectories are freely available at zenodo.org as https://doi.org/10.5281/zenodo.4050117.

## CRediT authorship contribution statement

**Kosala N. Amarasinghe:** Conceptualization, Formal analysis, Investigation, Writing – original draft. **Diana Pelizzari-Raymundo:** Formal analysis, Investigation, Methodology, Writing – review & editing. **Antonio Carlesso:** Conceptualization, Methodology, Supervision, Writing – original draft, Writing – review & editing. **Eric Chevet:** Conceptualization, Funding acquisition, Methodology, Writing – review & editing. **Leif A. Eriksson:** Conceptualization, Funding acquisition, Methodology, Supervision, Writing – review & editing. **Sayyed Jalil Mahdizadeh:** Conceptualization, Formal analysis, Investigation, Methodology, Supervision, Writing – original draft, Writing – review & editing.

## Declaration of Competing Interest

EC and LAE are co-founders of Cell Stress Discoveries, Ltd. EC is founder of Thabor Therapeutics. The authors declare no conflicting interests.
