# Supplementary material for: Sensor dimer disruption as a new mode of action to block the IRE1-mediated unfolded protein response
Source: Comput Struct Biotechnol J. 2022 Mar 29;20:1584–92. doi: 10.1016/j.csbj.2022.03.029 (PMC9010685; doi:10.1016/j.csbj.2022.03.029)
Supplement: Supplementary data 1 [file mmc1.pdf]

Sensor Dimer Disruption as a new Mode of Action to block the IRE1-mediated Unfolded Protein Response

Kosala N. Amarasinghe[1], Diana Pelizzari-Raymundo[2,3], Antonio Carlesso[1,4], Eric Chevet[2,3] Leif A. Eriksson[1,*] and Sayyed Jalil Mahdizadeh[1,**]

[1]Department of Chemistry and Molecular Biology, University of Gothenburg, 405 30 Göteborg, Sweden

[2]INSERM U1242, Université de Rennes, Rennes, France.

[3]Centre de Lutte contre le Cancer Eugène Marquis, Rennes, France.

[4] Università della Svizzera italiana (USI), Faculty of Biomedical Sciences, Euler Institute, Lugano, Switzerland

Correspondence:

*Leif A. Eriksson; email: leif.eriksson@chem.gu.se

**Sayyed Jalil Mahdizadeh; email: sayyed.jalil.mahdizadeh@gu.se

Supporting Information

Table S1 p.2

Figure S1 p.2

Figure S2 p.3

Figure S3 p.3

Figure S4 p.4-5

Figure S5 p.6

Figure S6 p.7

Figure S7 p.8-9

Figure S8 p.10

Figure S9 p.11

Figure S10 p.12

Figure S11 p.13

Figure S12 p.14

Table S1. The best four peptide sequences along with their docking scores and free energy of binding, ΔG_b, values.

Peptide sequence	Residue sequence[a]	Docking score (kcal mol^{-1})	ΔG_b (kcal mol^{-1})
**PFFWS**	830-834	-11.4	-76.6
**LRKFR**	886-890	-11.4	-72.0
**KKHHY**	907-911	-12.2	-62.2
**LFQPY**	956-960	-11.4	-59.9

[a]Residue sequence refers to the numbering in *h*IRE1.

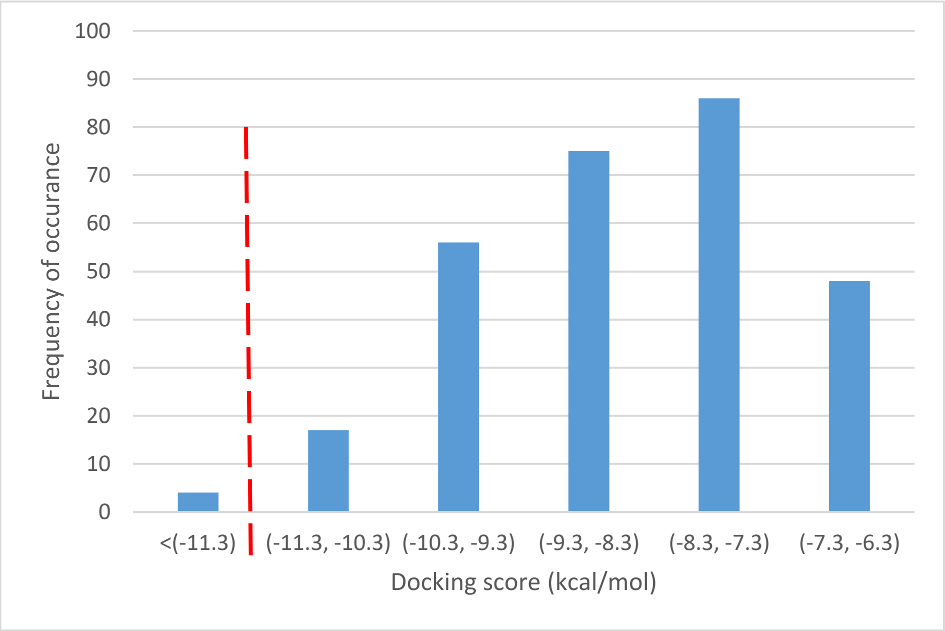

Figure S1. Frequency of abundance of the different IRE1 RNase sequence based peptides, in different docking score intervals (kcal/mol). The red line indicates the four peptides (docking score <(-11.3) kcal/mol) used in the pharmacophore modeling.

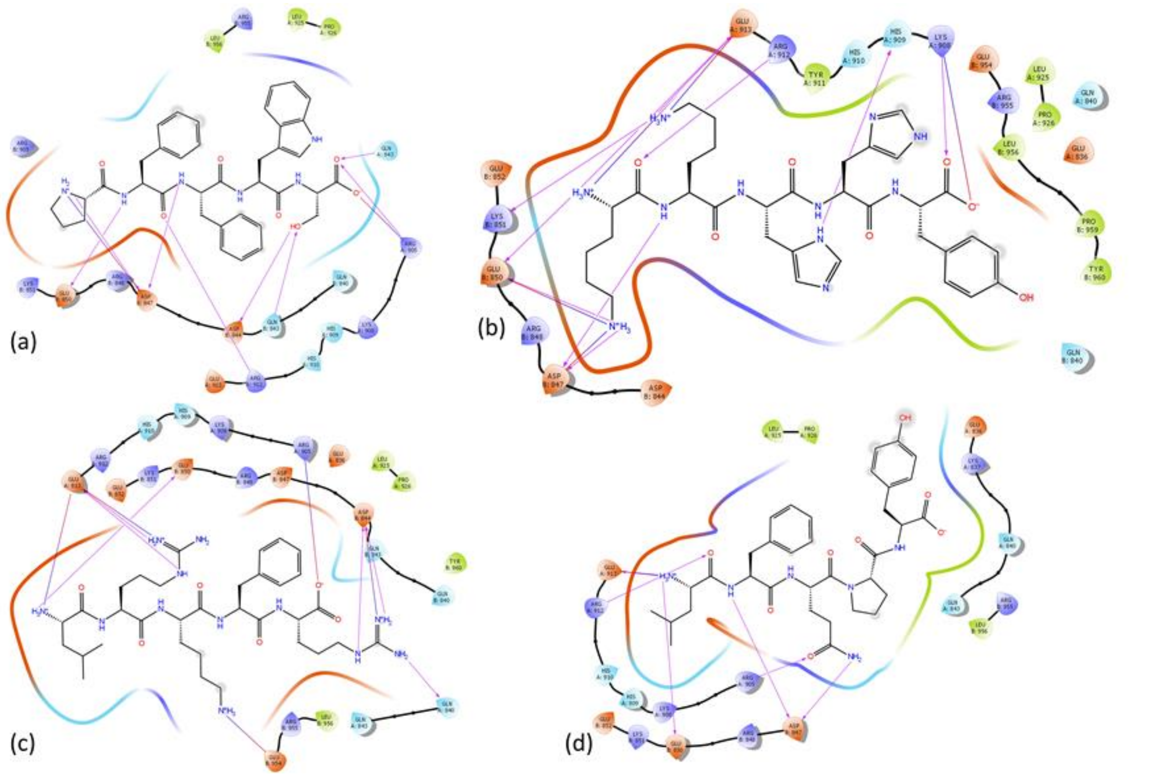

Figure S2. 2D interaction diagrams of the best peptide poses from the peptide docking calculations. (a) PFFWS, (b) KKHHY, (c) LRKFR, and (d) LFQPY.

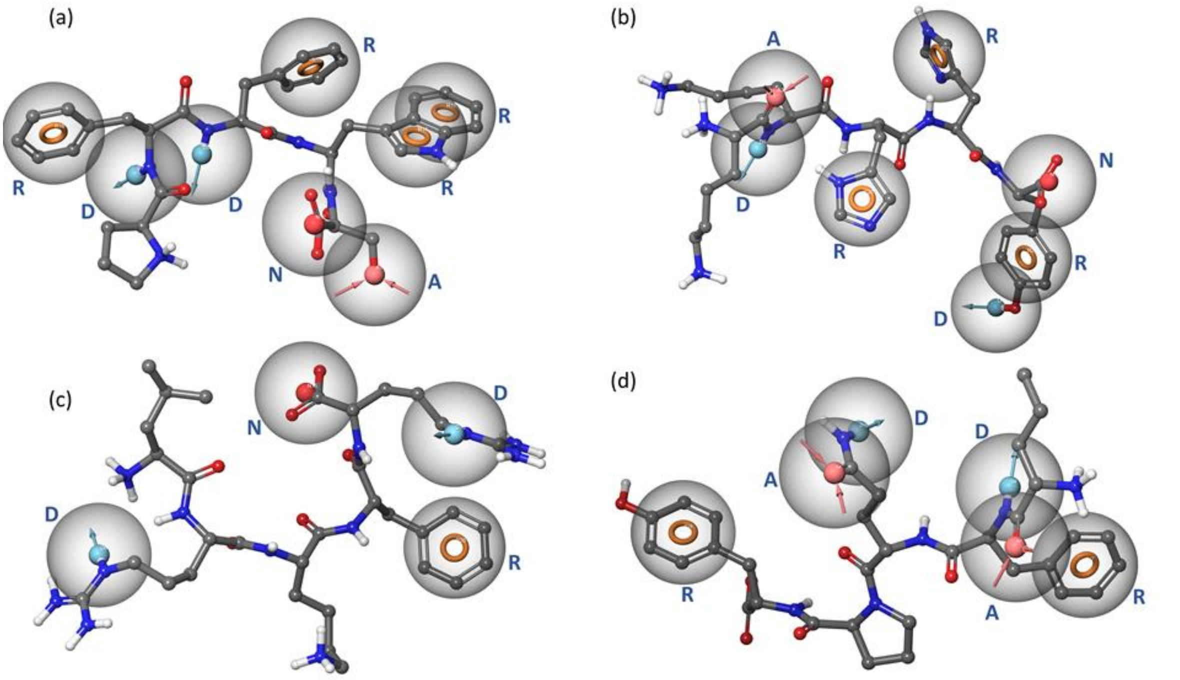

Figure S3. E-Pharmacophore hypotheses superposed on each peptide. (a) PFFWS, (b) KKHHY, (c) LRKFR, and (d) LFQPY. A, D, N, R refers to H-bond acceptor, H-bond donor, negative ionic site, and aromatic ring interaction, respectively, in the pharmacophore models.

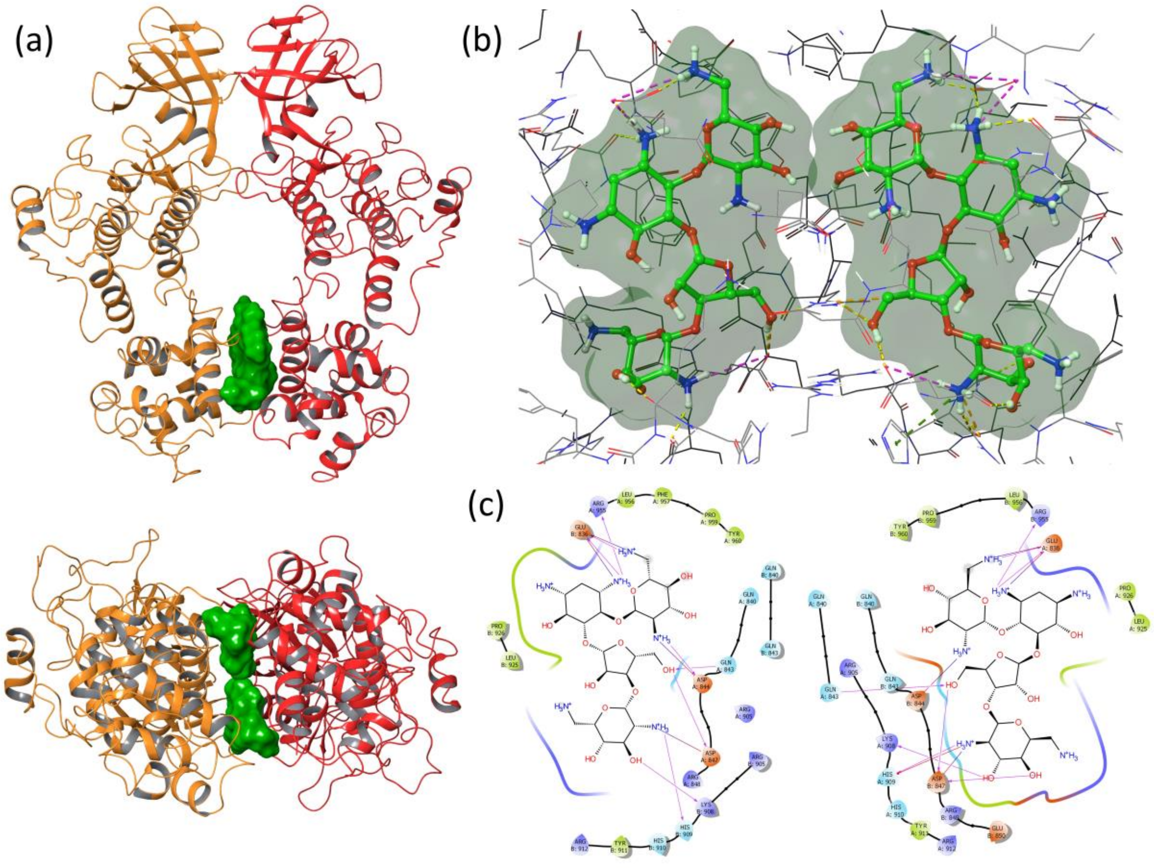

Chart I: Neomycin

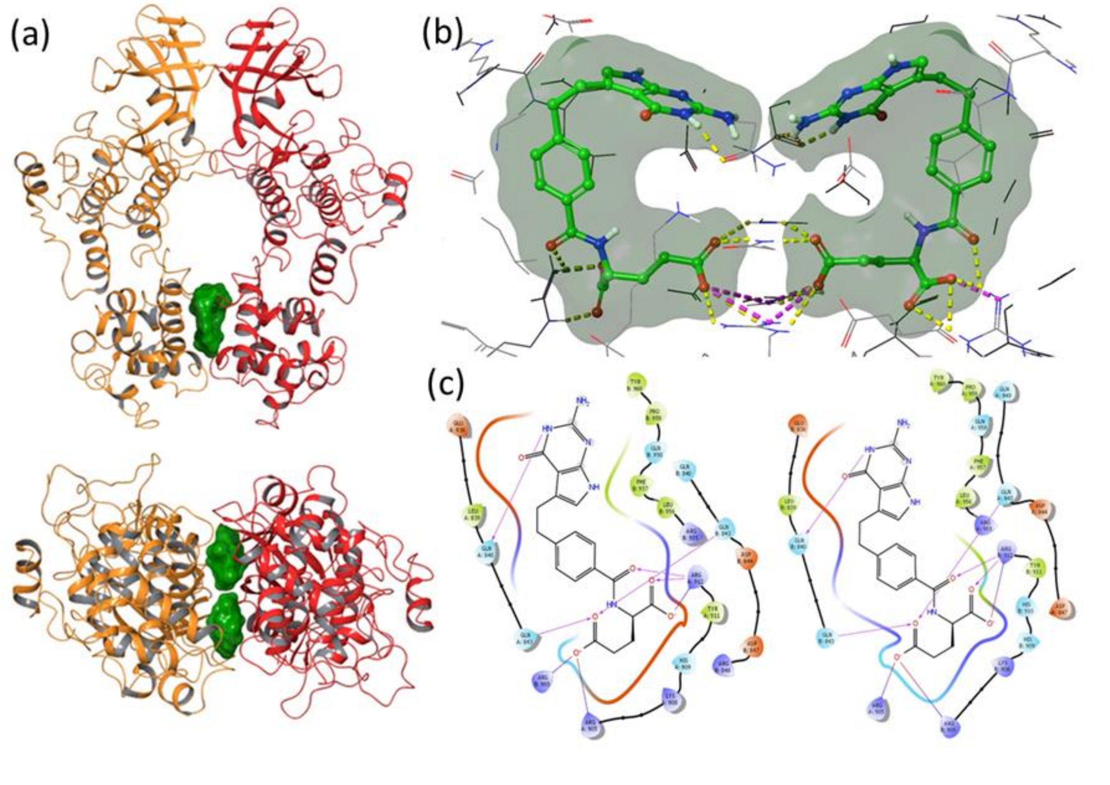

Chart II: Pemetrexed

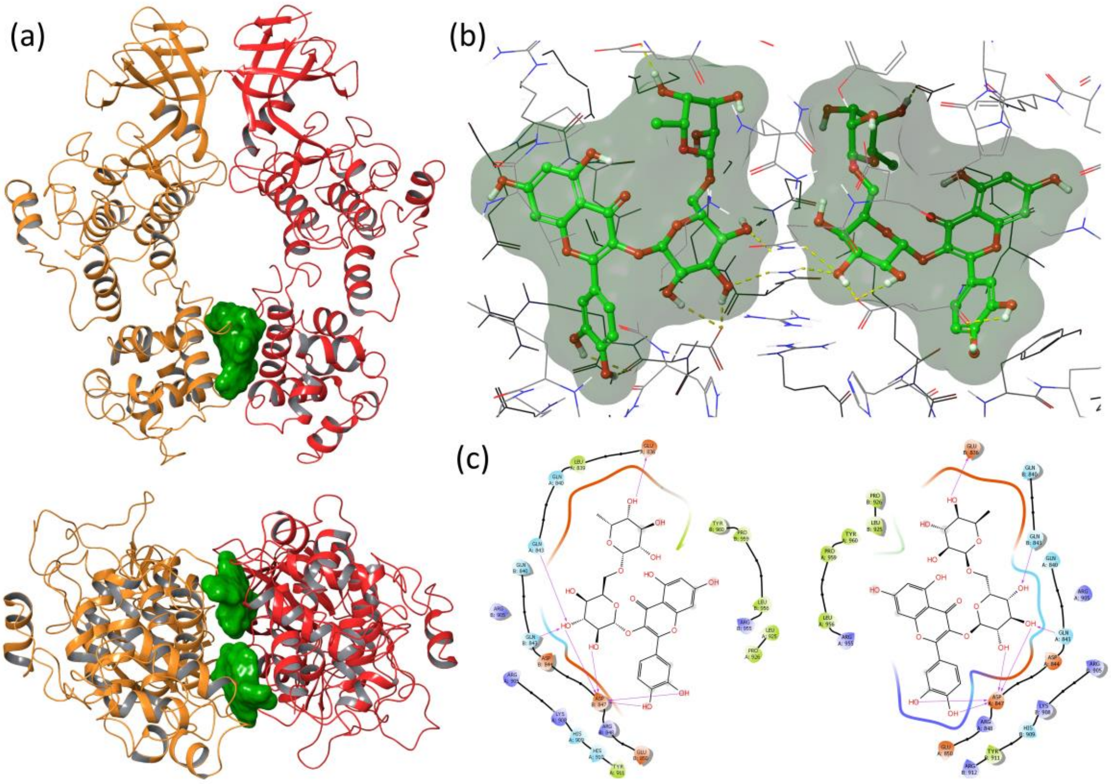

Chart III: Rutin

Figure S4. Chart I: Neomycin; Chart II: Pemetrexed; Chart III: Rutin. (a) 3D views of two molecules bound to the RNase domain of the IRE1 dimer from the molecular docking; top: front view; bottom: RNase domain viewed from below. One IRE1 monomer is depicted in red, the other in orange. (b) The corresponding atomic contacts in 3D. (c) 2D interaction maps. The corresponding data for quercitrin is displayed in Figure 4 in the main text.

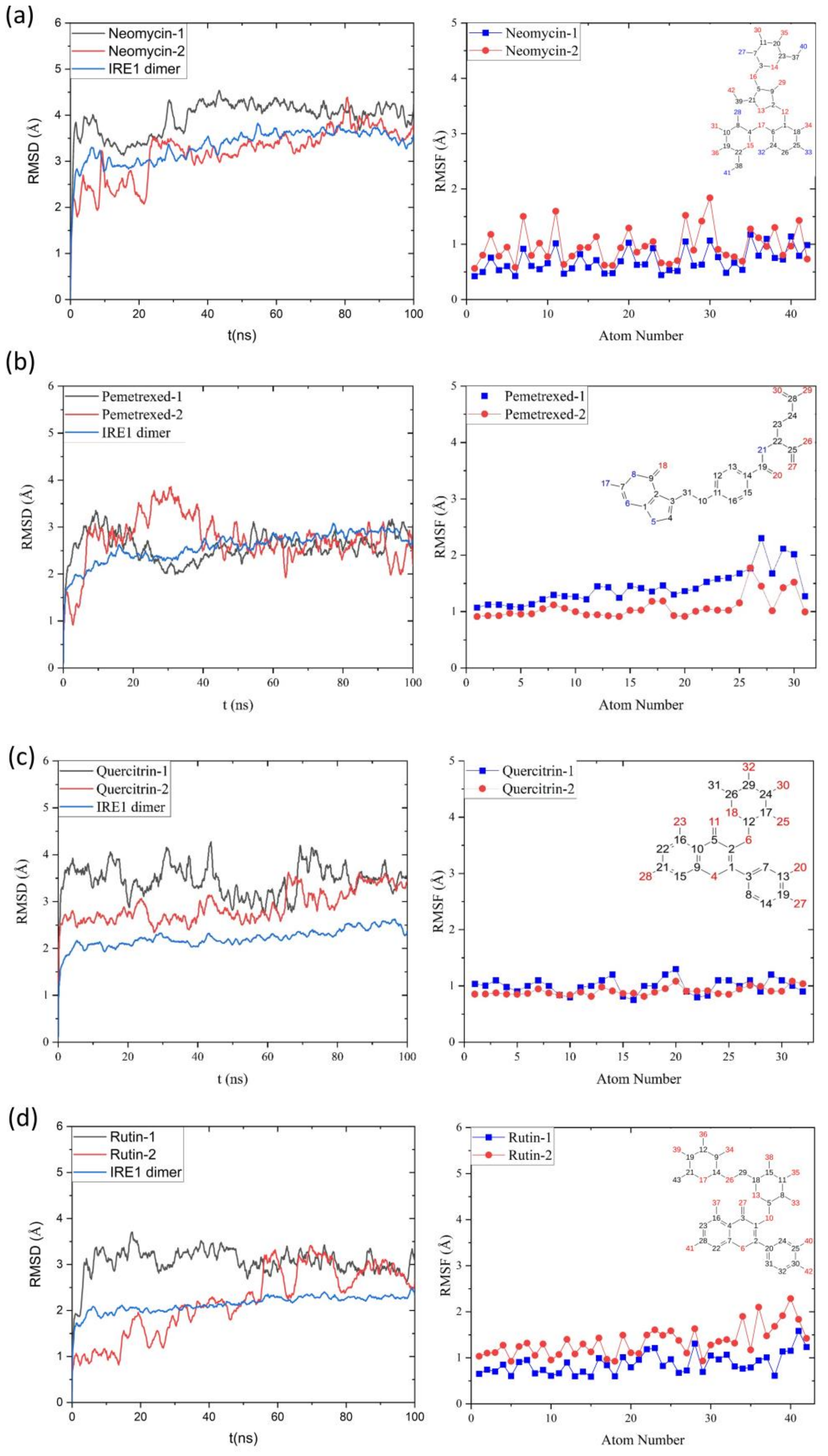

Figure S5. The RMSD and ligand RMSF plots of (a) Neomycin; (b) Pemetrexed, (c) Quercitrin and (d) Rutin molecules and IRE1 dimers during the 100 ns MD simulations. The atom numbers in the RMSF graphs are according to the 2D structure of the ligands presented in the inset panel.

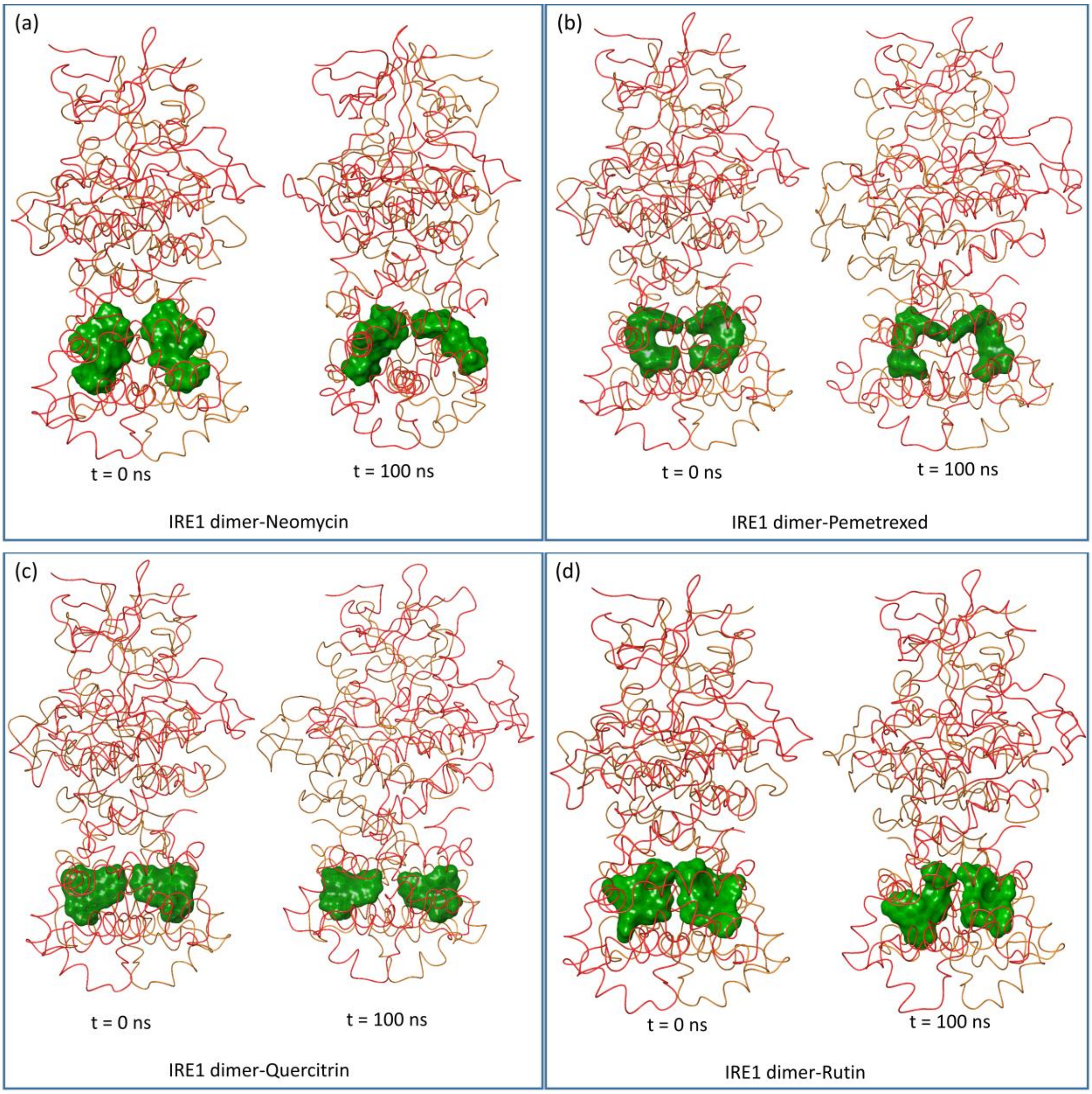

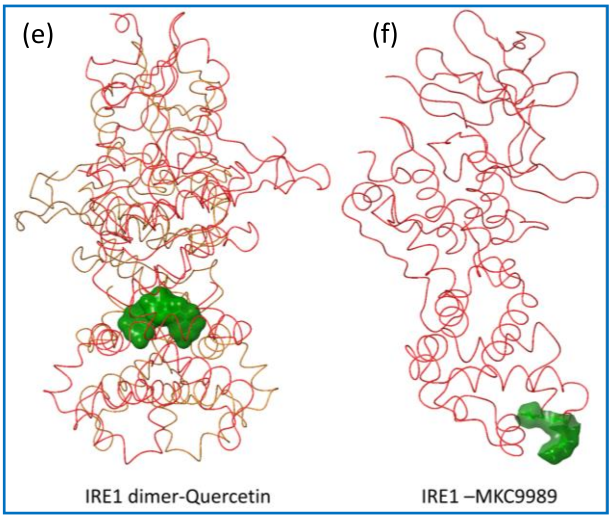

Figure S6. (a) to (d): side views of the first (t = 0 ns) and last (t = 100 ns) snapshots of hIRE1 dimer complexes during the MD simulations. (a) Neomycin, (b) Pemetrexed; (c) Quercitrin; (d) Rutin. (e) Activator quercetin bound to yIRE1 (PDB ID 3LJ0). (f) MKC9989 inhibitor covalently bound to murine IRE1 dimer (PDB ID 4PL3).

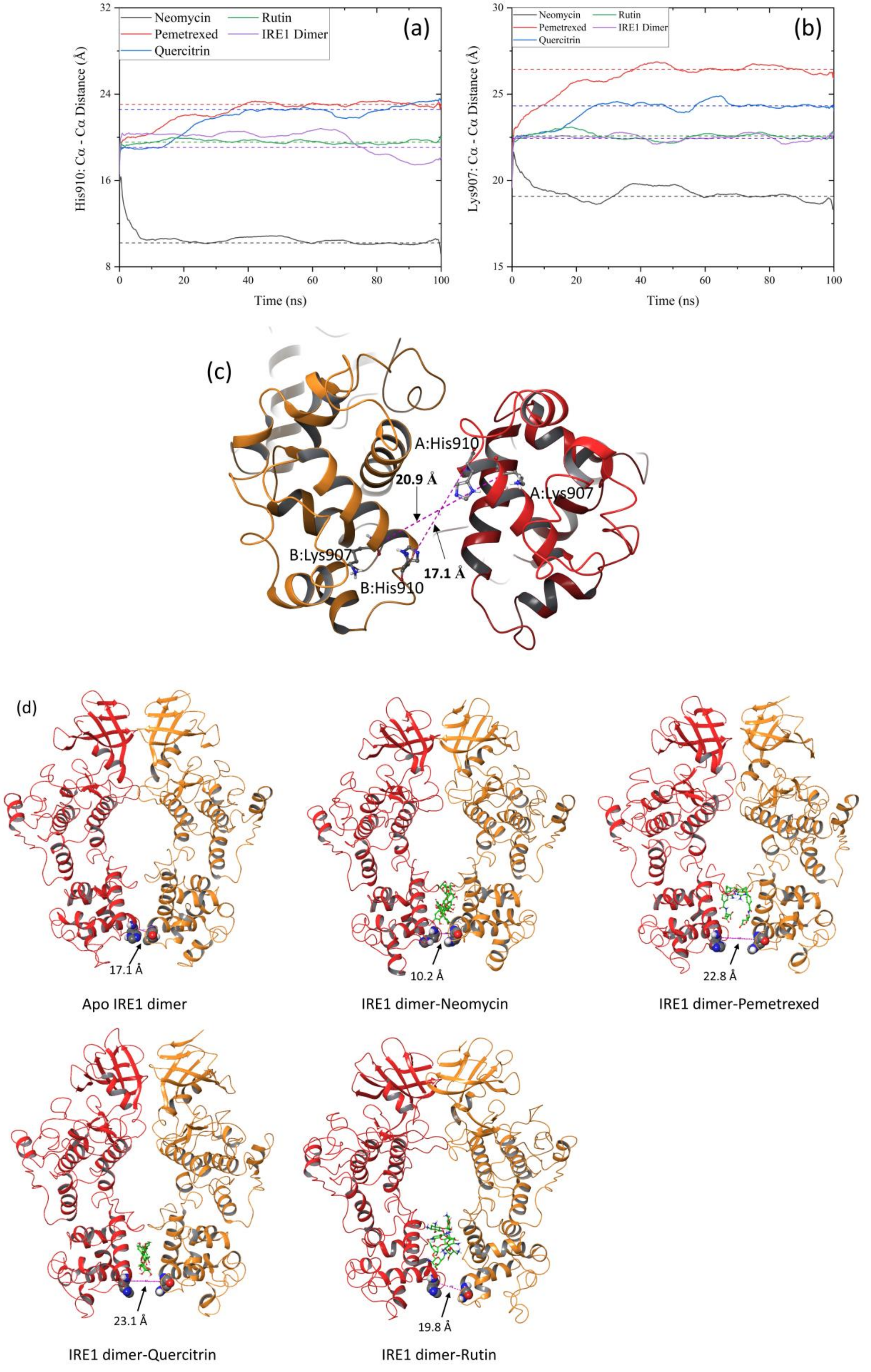

(a)
Neomycin
Rutin
Pemetrexed
IRE1 Dimer
Quercitrin
His910: Cα - Cα Distance (Å)
Time (ns)
(b)
Lys907: Cα - Cα Distance (Å)
(c)
A:His910
20.9 Å
A:Lys907
B:Lys907
17.1 Å
B:His910
(d)
17.1 Å
Apo IRE1 dimer
10.2 Å
IRE1 dimer-Neomycin
22.8 Å
IRE1 dimer-Pemetrexed
23.1 Å
IRE1 dimer-Quercitrin
19.8 Å
IRE1 dimer-Rutin

Figure S7. Distance between Cα atoms of active residues (a) A:His910 – B:His910 and (b) A:Lys907 – B:Lys907 during the MD simulations. The horizontal dashed lines indicate the average distances over the last 50 ns. (c) Distances between Cα atoms of residues His910 and Lys907 of the two monomers in the apo dimer (d) Conformational changes in the RNase domain of the IRE1 back-to-back dimer in apo form (PDB ID 4YZC) and bound to neomycin, pemetrexed, quercetin and rutin after 100 ns MD simulation. The pink dashed line shows the distance between Cα atoms of active residues A:His910 and B:His910 (both represented in space-filling model).

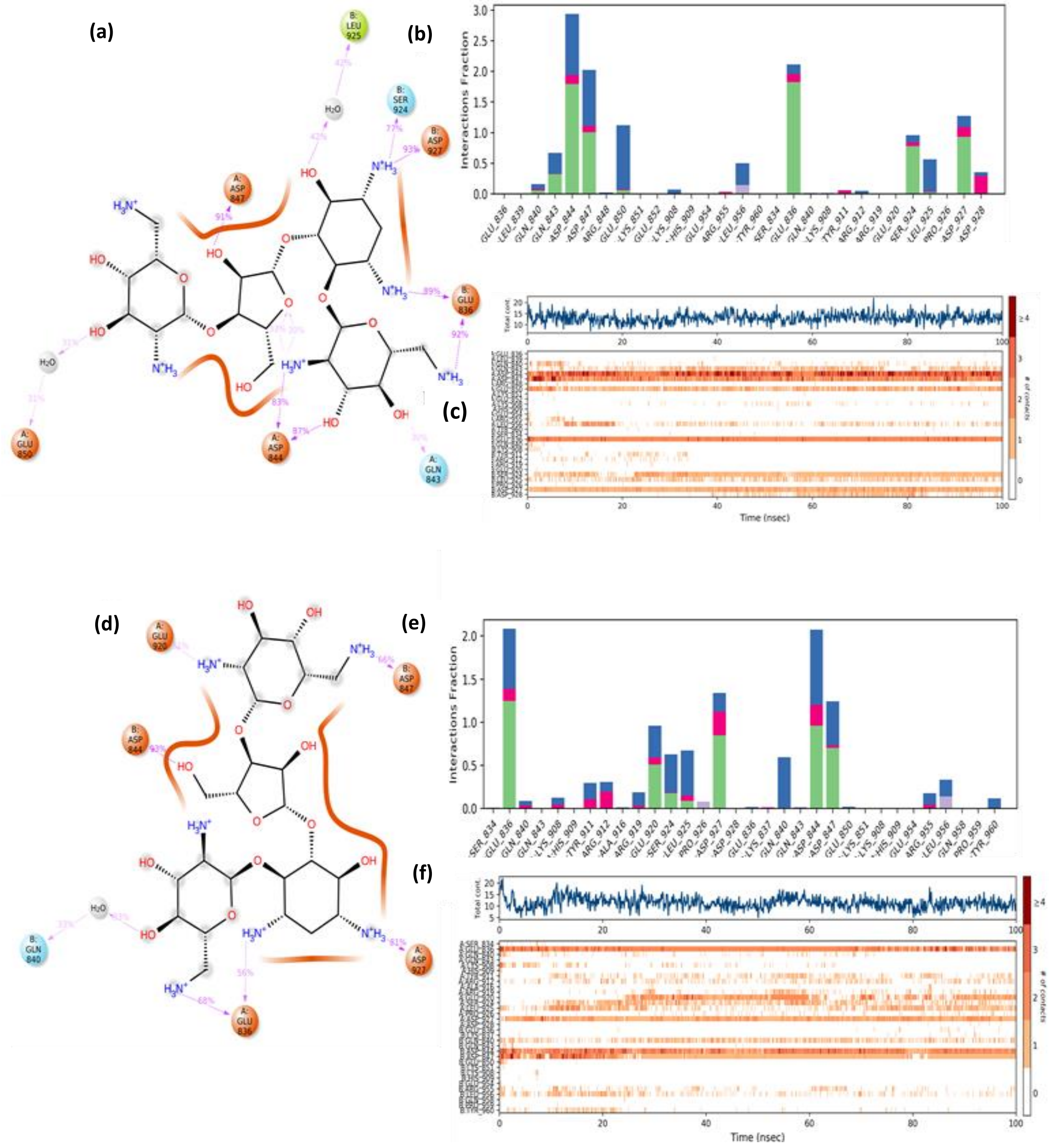

Figure S8. Abundance of protein–ligand interactions during 100 ns MD simulation for neomycin-1 (a) and neomycin-2 (d) along with corresponding histogram (b, e) and timeline (c, f) interaction diagrams.

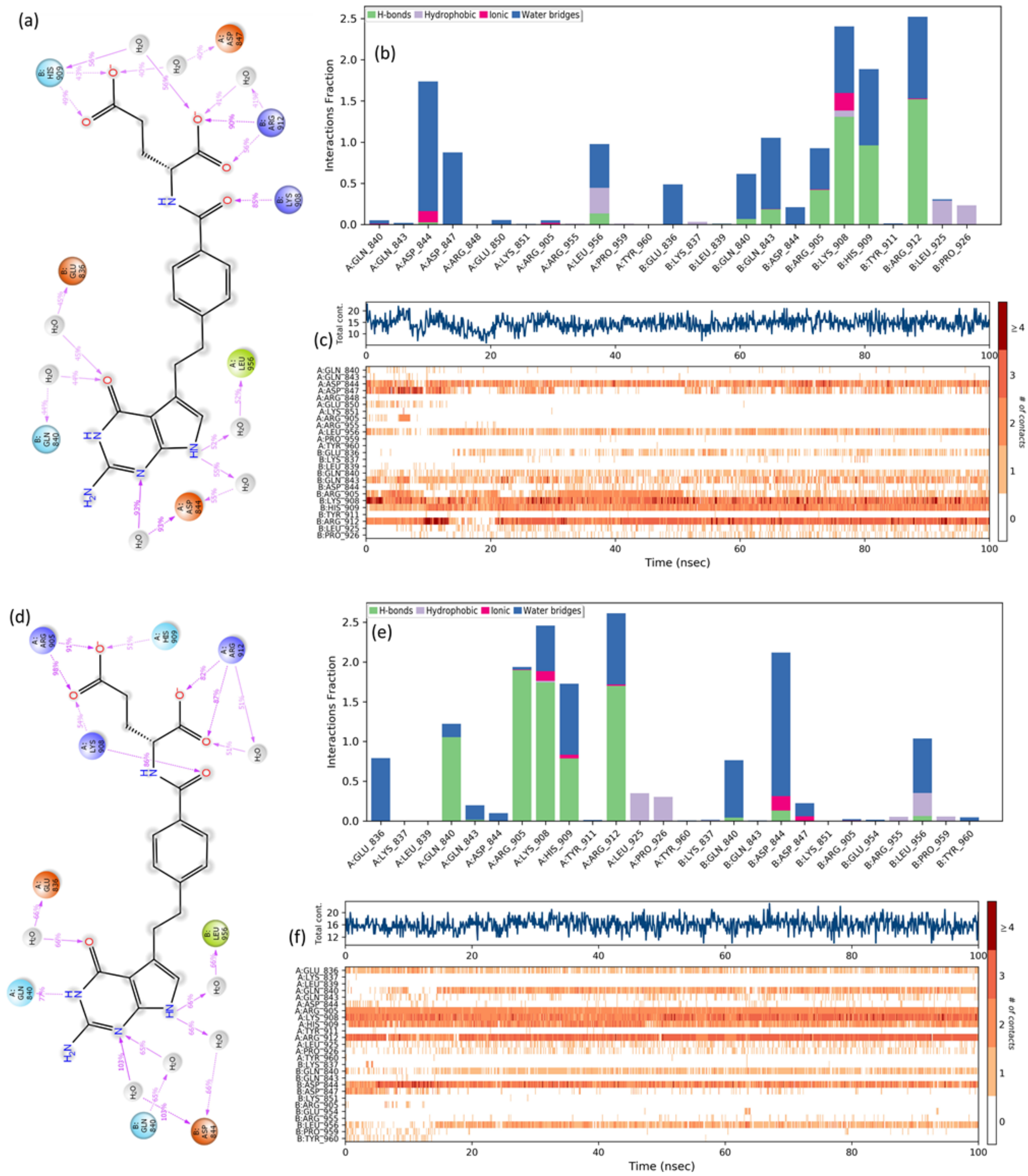

Figure S9. Abundance of protein–ligand interactions during 100 ns MD simulation for pemetrexed-1 (a) and pemetrexed-2 (d) along with corresponding histogram (b, e) and timeline (c, f) interaction diagrams.

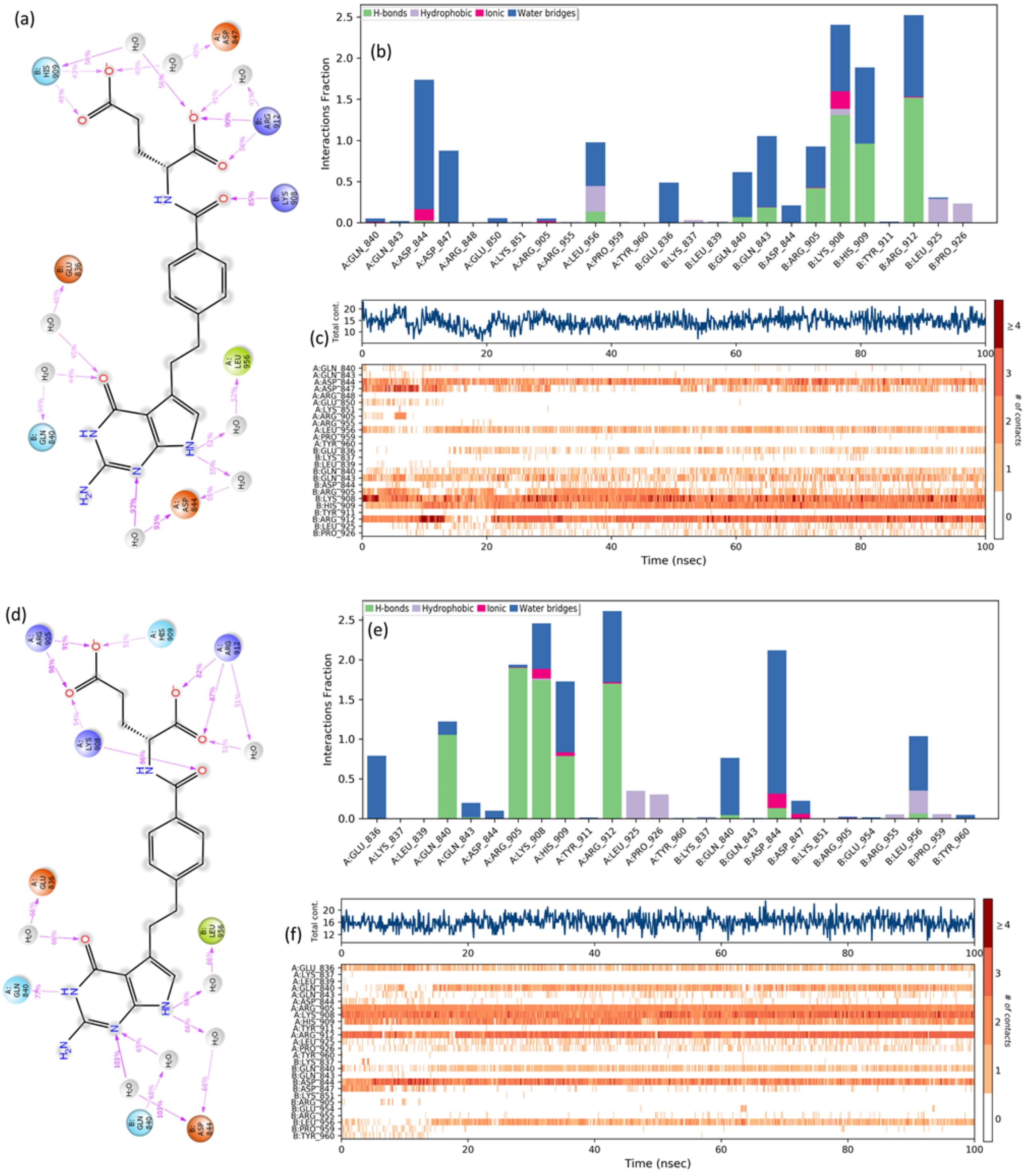

Figure S10. Abundance of protein–ligand atomic contacts during 100 ns MD simulation for quercitrin-1 (a) and quercitrin-2 (d) along with corresponding histogram (b, e) and timeline (c, f) interaction diagrams.

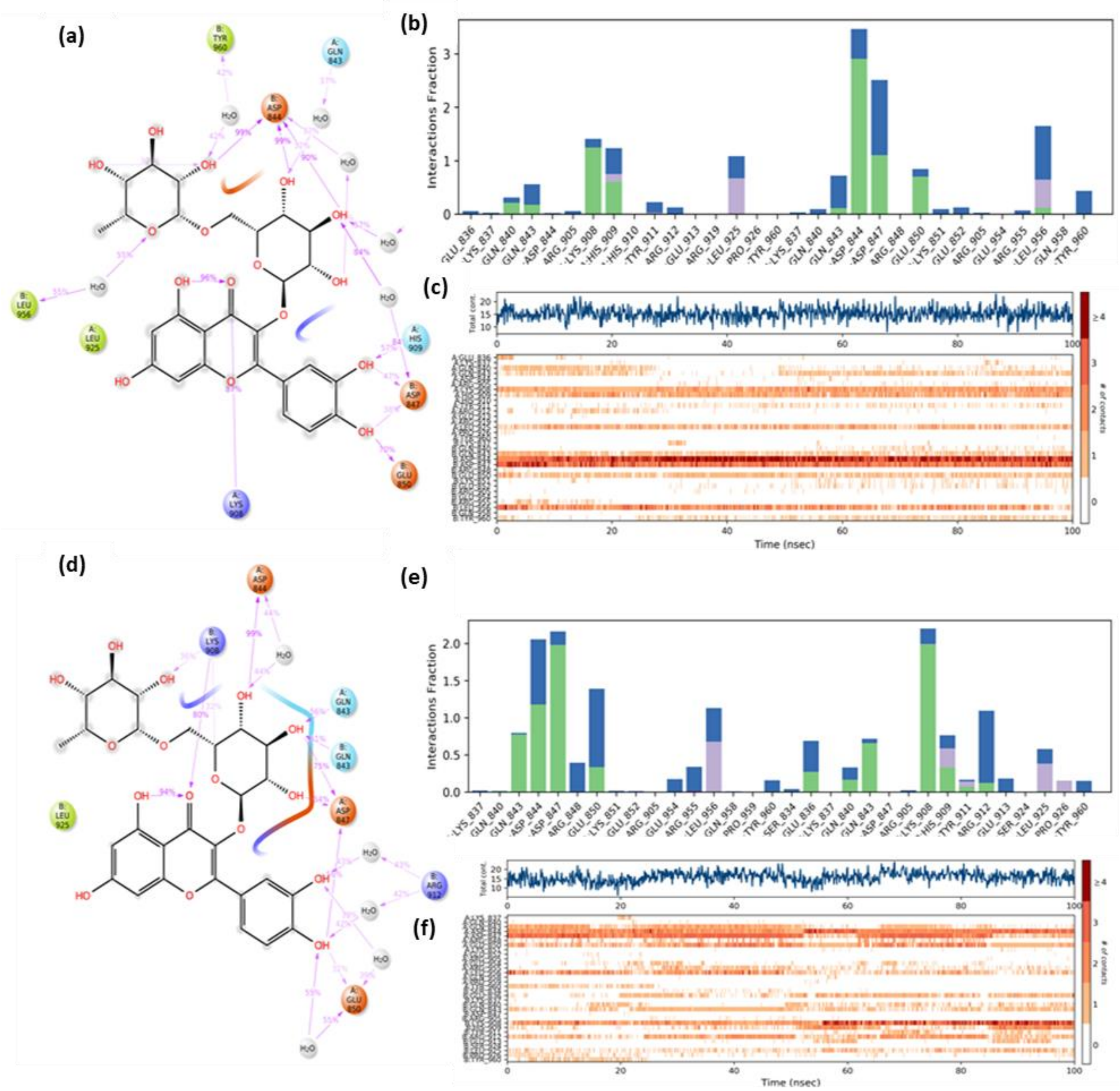

Figure S11. Abundance of protein–ligand atomic contacts during 100 ns MD simulation for rutin-1 (a) and rutin-2 (d) along with corresponding histogram (b, e) and timeline (c, f) interaction diagrams.

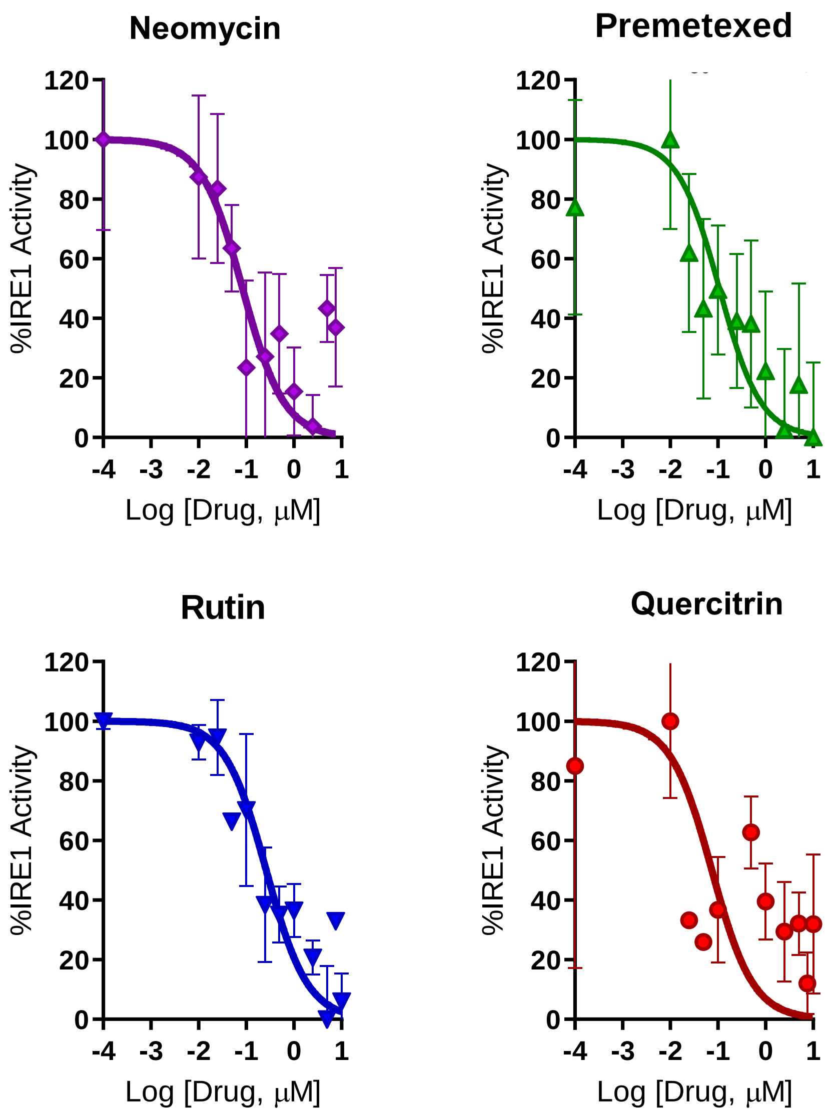

Figure S12. *In vitro* IRE1 RNase assay. Fitting curves of IRE1 RNase activity in the presence of increasing concentrations of neomycin**,** pemetrexed, rutin and quercitrin. Fluorescence signals were detected as a read-out of RNA probe cleavage after 25-minute incubation. Symbols and error bars represent mean values ± SEM.
